# Effect of Thermal History on Microstructures and Mechanical Properties of AZ31 Magnesium Alloy Prepared by Friction Stir Processing

**DOI:** 10.3390/ma7031573

**Published:** 2014-02-28

**Authors:** Fang Chai, Datong Zhang, Yuanyuan Li

**Affiliations:** National Engineering Research Center of Near-net shape Forming for Metallic Materials, South China University of Technology, Guangzhou 510640, Guangdong, China; E-Mails: chaifang-8806@163.com (F.C.); mehjli@scut.edu.cn (Y.L.)

**Keywords:** AZ31 magnesium alloy, friction stir processing, thermal history, microstructure, mechanical properties

## Abstract

Hot-rolled AZ31 (Mg-2.57Al-0.84Zn-0.32Mn, in mass percentage) magnesium alloy is subjected to friction stir processing in air (normal friction stir processing, NFSP) and under water (submerged friction stir processing, SFSP). Thermal history of the two FSP procedures is measured, and its effect on microstructures and mechanical properties of the experimental materials is investigated. Compared with NFSP, the peak temperature during SFSP is lower and the duration time at a high temperature is shorter due to the enhanced cooling effect of water. Consequently, SFSP results in further grain refinement, and the average grain size of the NFSP and SFSP specimens in the stir zone (SZ) are 2.9 μm and 1.3 μm, respectively. Transmission electron microscopy (TEM) examinations confirm that grain refinement is attributed to continuous dynamic recrystallization both for NFSP and SFSP. The average Vickers hardness in the SZ of the NFSP and SFSP AZ31 magnesium alloy are 76 HV and 87 HV. Furthermore, the ultimate tensile strength and the elongation of the SFSP specimen increase from 191 MPa and 31.3% in the NFSP specimen to 210 MPa and 50.5%, respectively. Both the NFSP and SFSP alloys fail through ductile fracture, but the dimples are much more obvious in the SFSP alloy.

## 1. Introduction

Magnesium alloys have been used in the aerospace, aircraft, and automobile industries due to their low density and high specific strength. In addition, magnesium alloys have excellent damping capability, good electrical and thermal conductivity, and recyclability [1,2]. However, the ductility of magnesium alloys at room temperature is generally not good due to their HCP structure. This is one of the main limitations for expanding the application of magnesium alloys. Consequently, it is necessary to improve the formability of magnesium alloy.

It is well-known that grain refinement is an effective method to improve the ductility of magnesium alloys. In the past few years, fine-grained magnesium alloys have been successfully produced by severe plastic deformation (SPD) techniques, such as equal channel angular pressing (ECAP) [3–5], accumulative roll-bonding (ARB) [6], and high pressure torsion (HPT) [7]. Recently, friction stir processing (FSP), proposed by Mishra *et al.* [8–10] based on the principles of friction stir welding (FSW), has drawn great interests due to its potential in fine or ultrafine-grained metallic materials preparation. As a novel SPD technique, FSP is used to refine the microstructures of magnesium alloys and consequently improve their ductility. Table 1 summarizes the grain size and mechanical properties of AZ31 magnesium alloys prepared by FSP [11–18]. As can be seen in Table 1, with the grain size decreasing, mechanical properties of the FSP AZ31 alloys generally increase. The average grain size of the FSP AZ31 magnesium alloys varies in the range of 2–13 μm. On the other hand, some research has been conducted on ECAP of AZ31 magnesium alloy, and much finer grain structures are produced with this method. For instance, Jim *et al.* [19] produced an ultrafine-grained AZ31 alloy with an average grain size of 0.5 μm through two-passes ECAP. A fine-grained AZ31 magnesium (1.7 ± 0.2 μm) alloy was prepared by four-passes ECAP at 200 °C with a yield tensile strength of 118 MPa, an ultimate tensile strength of 244 MPa and an elongation of 26.2% [20]. ECAP, as a typical SPD technique, has been proved successfully in producing much finer-grained AZ31 alloy with generally much higher tensile strength in comparison with FSP. However, at least four to six passes of ECAP are generally needed to achieve grain refinement due to the low efficiency.

It is widely accepted that the combination of FSP with rapid cooling is an effective method to achieve grain refinement and mechanical properties improvements [8]. Chang *et al.* developed an efficient cooling system, in which a copper mold, full of liquid nitrogen, was used as rapid heat sink during FSP. On this novel equipment, they successfully produced ultrafine-grained AZ31 magnesium alloys with an average grain size of 100–300 nm [21] and nano-grained AZ31 alloy [22]. Du *et al.* [23] prepared ultrafine-grained microstructures with an average size of about 300 nm in AZ61 magnesium alloy using FSP combined with liquid nitrogen. Su *et al.* [24] prepared 7075Al with an average grain size of ~100 nm via FSP, using a mixture of water, methanol, and dry ice to cool the plate rapidly. In recent years, submerged friction stir processing (SFSP) comes up as a new variation to FSP, which means the entire processing of the plate is done underwater. Due to its high specific heat capacity, water can absorb a large amount of frictional heat and lead to a high cooling rate. Compared with other cooling systems, SFSP is convenient, economical and environmental-friendly. Tokisue *et al.* [25] firstly used submersion in a friction joining process, and they successfully joined 6061 aluminum alloy underwater. Liu *et al.* [26] conducted water-submerged FSP on the 2219-T6 aluminum alloy, and the grain size was greatly refined from 17 μm to 1.3 μm. The study of Hofmann *et al.* [27] showed that SFSP was a way of increasing the cooling rate of the bulk samples and decreasing the grain size. To present, SFSP is a relatively new technique, and most of the researches are confined to aluminum alloys. Limited numbers of studies have been conducted in the field of magnesium alloys prepared by SFSP, especially on the measurement of thermal history. Darras *et al.* [28] conducted FSP of AZ31 magnesium alloy in air, hot water, and cold water, and measured the temperature during processing; the finest microstructure with an average grain size of 13.3 μm was gained in the FSP specimen submerged in cold water, and the highest elongation (18.3%) was achieved when the specimen was processed in hot water. However, the microstructure evolution has not been discussed in detail in their study.

In this study, the hot-rolled AZ31 magnesium plate was subjected to FSP in air and under water, respectively. For the convenience of statement, FSP performed in air is defined as normal FSP (NFSP). Thermal histories of the two FSP procedures were recorded, and the effect of temperature on the final microstructure and mechanical properties was investigated.

## 2. Results

### 2.1. Temperature Profile

**Figure 1.**
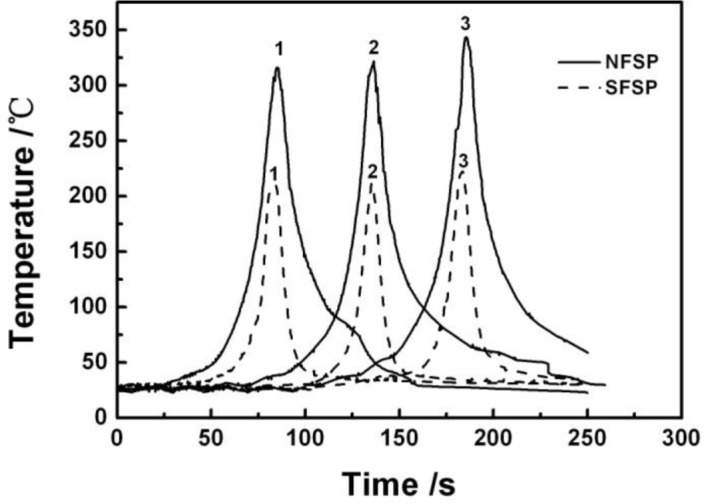
Temperature profiles during FSP of AZ31 magnesium alloy (1, 2, and 3 are the locations mentioned in Figure 11).

Figure 1 shows temperature profiles of the two FSP procedures, the different curves corresponding to the locations marked as 1, 2, and 3, marked in Figure 11. As can be seen in the figure, the temperature curves during NFSP and SFSP show the same trend at the three positions, *i.e*., the temperature increases from low temperature as the pin moves and reaches the highest value when the pin arrives at the measured position, and then decreases with the departure of the pin. Both for NFSP and SFSP, the peak temperature at position 3 is the highest, and the peak temperatures at position 1 and 2 are almost the same. However, the peak temperatures of the two FSP procedures are quite different. The peak temperatures at the position 1, 2, and 3 during NFSP are 316 °C, 322 °C, and 344 °C, respectively. For SFSP, the peak temperatures at the three positions are much lower than those of the NFSP, and the peak temperatures are 211 °C, 212 °C, and 223 °C, respectively. That is to say, the peak temperature of SFSP is 100–120 °C lower than that of NFSP. Compared with NFSP, the temperature cools down more rapidly during SFSP, thus, the temperature curve is steeper and the duration time at high temperature is shorter for SFSP. For example, when the temperature is above 150 °C, the duration time is 27 s for NFSP while the time decreases to 8 s for SFSP, indicating a higher cooling rate can be achieved during SFSP.

### 2.2. Microstructure

**Figure 2.**
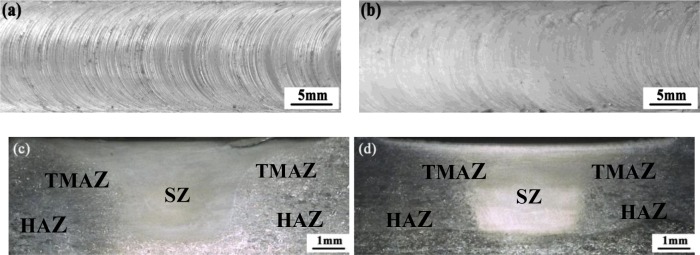
Top surface morphology (**a**) in air and (**b**) underwater, and macro-structures of the cross-section (**c**) in air and (**d**) underwater of the FSP specimens.

Figure 2 shows the optical images of top surface and cross-section of the two FSP specimens. Three microstructural zones, namely thermo-mechanically affected zone (TMAZ), heat-affected zone (HAZ), and SZ, are identified. Annular ring patterns can be seen clearly on the top surface of the processed plates, and no defects, such as pores and cracks, are found in the NFSP and SFSP specimens. Comparing Figure 2a with b, one can observe that the top surface of the SFSP plate is much smoother than that of the NFSP plate. In addition, the SZ-areas are 12.7 mm^2^ and 11.8 mm^2^ for the NFSP and SFSP specimens measured by the Image-Pro-Plus software. That is mainly due to the lower temperature during SFSP (shown in the Figure 1), which leads to the amount of metal stirred by the pin reducing.

**Figure 3.**
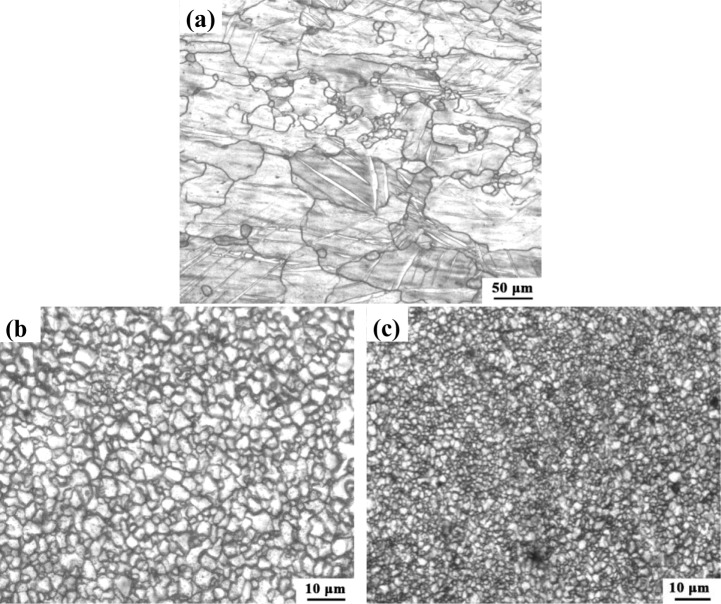
Microstructures of the experimental material: (**a**) BM; (**b**) SZ processed in air; (**c**) SZ processed underwater.

Figure 3a shows the OM microstructures of the hot-rolled AZ31 magnesium alloy. The BM exhibits a bimodal microstructure, which is mainly composed of coarse α-Mg grains (~102.7 μm) with some small grains (~7.8 μm) distributed in the matrix. Twins are also observed in the BM. Figure 3b,c show the microstructures of the SZ for AZ31 magnesium alloys prepared by NFSP and SFSP, respectively. The grains are effectively refined and distributed more homogeneously after FSP. Compared with NFSP, SFSP results in further grain refinement, and the average grain size of the NFSP and SFSP specimen is 2.9 μm and 1.3 μm, respectively.

**Figure 4.**
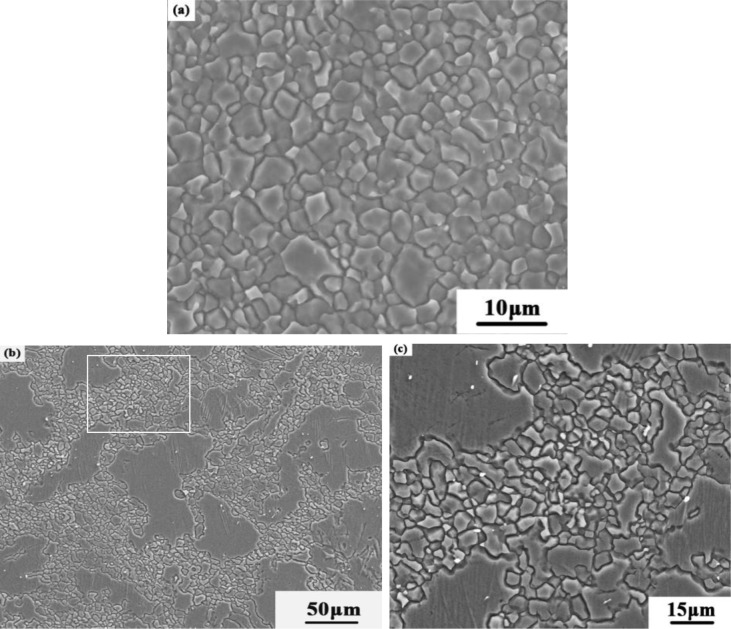
Representative grained structures of the NFSP AZ31 magnesium alloy: (**a**) SZ; (**b**) TMAZ; (**c**) High magnified image of the marked position in Figure 4b.

Figure 4 shows the representative SEM microstructures of the SZ and the TMAZ for the NFSP AZ31 magnesium alloy. Figure 4a, which shows a SEM-image of the microstructure in the SZ, illustrates the homogeneous and fine grain distribution typical of this portion of the material. The microstructure of the TMAZ is rather characterized by a bimodal population of some highly deformed grains and fine recrystallizated grains (Figure 4b). The magnified image of the marked position in Figure 4b is shown in Figure 4c. Some extremely fine grains (1.2–1.6 μm) are observed in the NFSP specimen.

**Figure 5.**
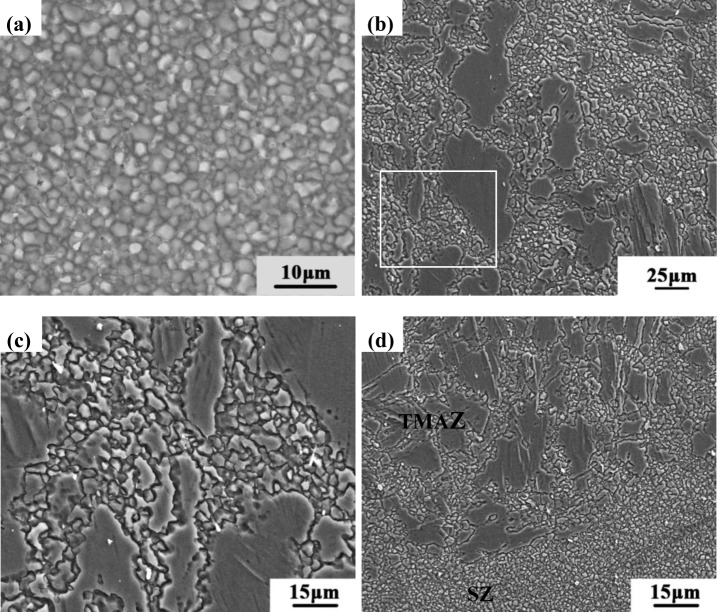
Representative SEM images of the SFSP AZ31 magnesium alloy: (**a**) SZ; (**b**) TMAZ; (**c**) High magnified image of the marked position in Figure 5b; (**d**) TMAZ/SZ.

Figure 5 shows the SEM microstructures of the SFSP AZ31 magnesium alloy. The SZ is characterized by fine equiaxed grains due to dynamic recrystallization (DRX) (Figure 5a). Figure 5b shows the microstructures in the TMAZ and the highly magnified image of the marked position is shown in Figure 5c. The TMAZ experiences partial DRX because the temperature is not high enough and the deformation is not as severe as in the SZ. Some ultra-fine grains with a size of 0.3–0.6 μm are distributed among the coarse and elongated grains. Figure 5d shows the SEM image of the interface between the SZ and the TMAZ. Different from the TMAZ, material in the SZ undergoes full DRX and subsequent grain growth. No coarse grains remain in the SZ, but the size of the newly-formed grains is a little larger than that of the ultra-fine grains in the TMAZ.

**Figure 6.**
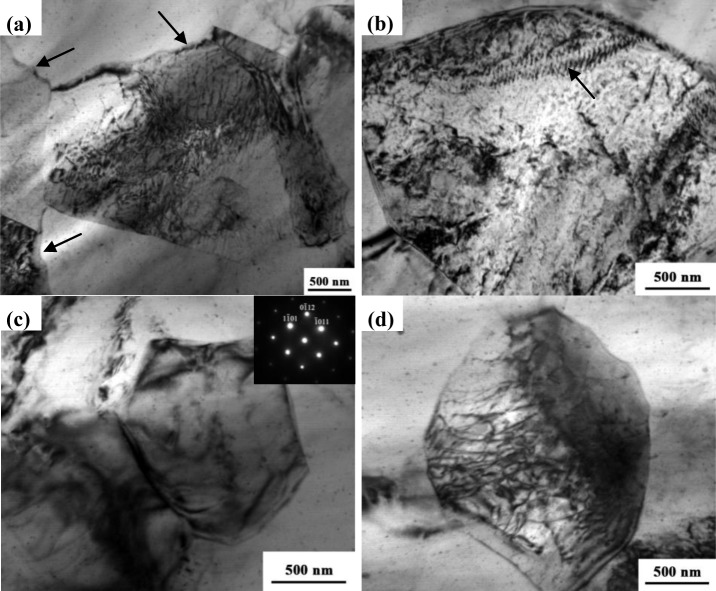
Typical TEM micrographs of the FSP AZ31 specimen: (**a**) and (**b**) NFSP; (**c**) and (**d**) SFSP.

Figure 6a,b show typical examples of TEM micrographs of the NFSP AZ31 magnesium alloy. The recrystallized grains together with the selected area diffraction (SAD) patterns are shown in Figure 6a. The recrystallized grains with serrated grain boundaries (shown by arrows) can be observed. In addition, dislocations pile up against the boundaries and dense dislocation walls are clearly visible in Figure 6b. Figure 6c,d present two examples of typical microstructures observed in the SFSP AZ31 alloy. Fine equiaxed grains resulting from DRX and SPD can be observed in Figure 6c. The average grain size is about 1.4 μm, which is in agreement with the SEM observation. Some dislocations and subgrain boundaries can be obviously observed in Figure 6d.

## 2.3. Mechanical Properties

**Figure 7.**
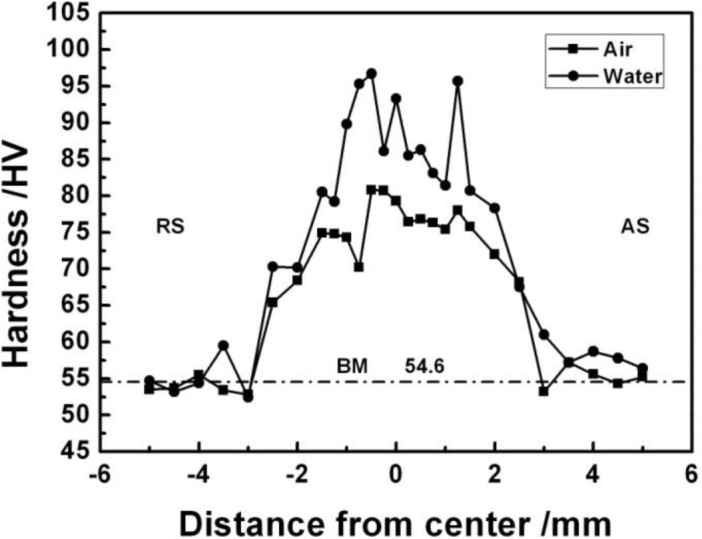
Microhardness distributions of the experimental materials.

Figure 7 shows the microhardness distribution of the experimental material on the transverse cross-section. The average hardness of the BM is about 55 HV. The hardness of the FSP specimen is the highest in the SZ and decreases at both sides. The hardness in the HAZ and TMAZ on the advancing side (AS) is a little higher than that on the retreating side (RS). Compared with NFSP and SFSP, the average hardness of the SFSP specimen is much higher than the NFSP specimen, which are 87 HV and 76 HV, respectively.

**Figure 8.**
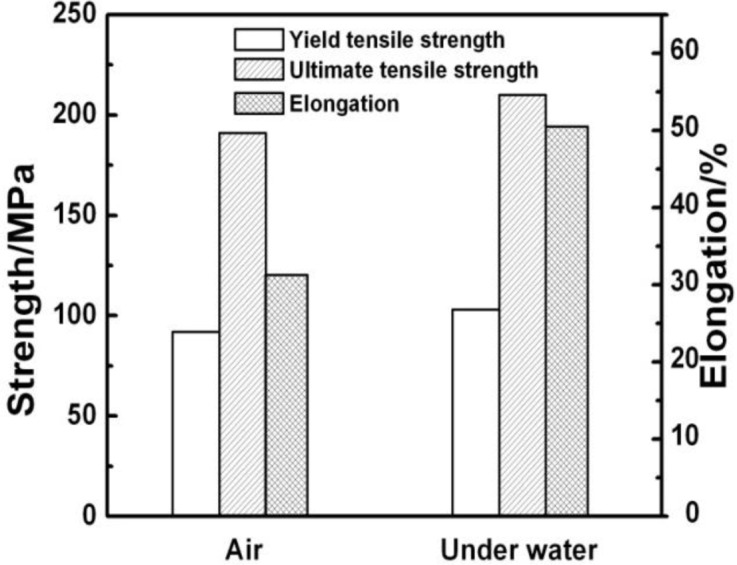
Tensile test result of the FSP AZ31 magnesium alloy processed in air and underwater.

Figure 8 summarizes the tensile properties of AZ31 magnesium alloy. As shown in Table 2, the BM exhibits an elongation of ~25% and an ultimate tensile strength of ~250 MPa. After FSP, the ultimate tensile strengths decrease while the elongations improve significantly. Hung *et al.* [14] reported that due to a change in grain orientation and retained stress induced by FSP, the refinement grains after FSP had no contribution to tensile strength. Woo *et al.* [29] also showed that a decreasing tensile strength in the FSP of AZ31 magnesium alloy was achieved compared to the BM, and they attributed it to the drastic texture variation during FSP. The yield tensile strength, ultimate tensile strength and elongation of the NFSP specimen are 92 MPa, 191 MPa, and 31.3%, respectively. As to SFSP, those properties are increased to 103 MPa, 210 MPa, and 50.5%. Namely, the tensile properties of the SFSP specimen are improved in comparison to the NFSP specimen, especially the ductility. The excellent ductility of the SFSP specimen is attributed to its fine microstructure achieved by enhancing cooling rate.

**Figure 9.**
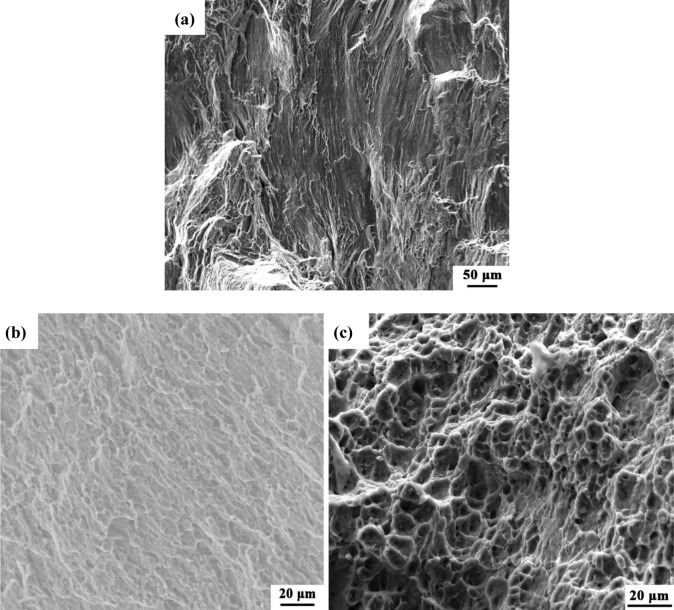
Fracture surfaces of AZ31 alloys at room temperature: (**a**) BM; (**b**) in air; (**c**) underwater.

Figure 9a is the fracture surface of the BM, which is characterized by cleavage steps, river patterns and a few dimples. Figure 9b,c shows the fracture surfaces of the NFSP and SFSP specimens, respectively. Shallow-hole type dimples are found in the NFSP specimens, while the dimples in the SFSP specimen are much deeper and distribute more uniformly. With the grain size decreasing, the ductility of the experimental material increases, and the ductile fracture feature becomes more obvious.

## 3. Discussion

### 3.1. Thermal History Analysis

The peak temperature during NFSP is about 354 °C, which is much lower than the melting point of AZ31 magnesium alloy (610 °C) [1], thus, no melting takes place during the process. Due to the cooling effect of water, the peak temperatures of SFSP are lower and the temperature distribution curves are steeper in comparison to NFSP, indicating that a higher cooling rate can be achieved during SFSP. In NFSP, the processing temperature drops from the peak temperature of ~327 °C to approximately 50 °C in about 70 s, while only 14 s are needed in SFSP. By a rough evaluation, the average cooling rate of NFSP and FSP is 4.0 °C/s and 11.8 °C/s, respectively. Darras *et al.* [28] reported that both the peak temperature and the time spent above a certain reference temperature reduced when FSP of AZ31 performed under water. A sharper peak and faster cooling rate was also observed during SFSP of 6061 aluminum alloys [30]. The peak temperatures, measured by five thermocouples, ranged between 278–406 °C and 418–469 °C, for SFSP and NFSP, respectively. In general, the peak temperature of SFSP is 60–140 °C lower than that of NFSP in magnesium and aluminum alloys. SFSP is similar to conducting FSP with on-line quench, in which the duration time at high temperature is very short for the newly-formed fine grains to grow coarse.

### 3.2. Effect of Thermal History on Grain Size

Thermal history of FSP plays an important role on the final grain size. Many papers have suggested that the equiaxed grains in the SZ are produced by DRX during the stirring of the material [31–33]. Li *et al.* [31] reported that dynamic recrystallized grains experienced static grain growth during the cooling of the plate, and the following equation for the static grain growth within the SZ should be satisfied:

$$D^{2}-D_{0}^{2}=A\text{exp}(-Q/RT)\;t$$ (1)

where *D* and *D*_0_ are the final and initial (recrystallized) FSW grain size, respectively, *A* is a constant, *Q* is the appropriate activation energy for grain growth, *R* is the ideal gas constant, *T* is the absolute temperature, and *t* is the time. Sato *et al.* [34] reported that the *D*_0_ value was significantly smaller than the *D* value, thus the *D*2 0 value could be ignored. Therefore, Equation (1) can be converted into the following equation:

$$\text{ln}D=\text{ln}\frac{At}{2}-\frac{Q}{2RT}$$ (2)

According to the equation, when the values of *T* and *t* are reduced, the grain size will be smaller. The study of Chang *et al.* [13] showed that the temperature ***T*** was the only one factor determined the resulting grain size when the dimensions of the tool and processing parameters were the same, and the lower the temperature resulted in the smaller the grain size. In our study, the thermal fields are very sensitive to the submerging conditions as shown in Figure 1. Compared with NFSP, the peak temperature (*T*) of SFSP is lower, and the time (*t*) that the SFSP specimen spent at high temperature is shorter. Consequently, much finer-grained microstructures can be achieved by SFSP.

### 3.3. DRX Mechanisms

It is widely accepted that DRX takes place during FSW/FSP, resulting in generation of fine and equiaxed grains in the SZ [8]. DRX occurs readily in Mg and its alloy due to the following reasons: (1) lack of easily activated slip system; (2) high boundary diffusion rate; and (3) relatively low stacking fault energy [35]. Three mechanisms are proposed for DRX in the FSW/FSP of magnesium alloy, namely continuous DRX (CDRX), discontinuous DRX (DDRX), and twinning DRX (TDRX) [22,36]. CDRX is characterized by subgrain rotation without recognizable “nucleation” and “growth” of the recrystallized grains, and the microstructure evolves homogeneously. On the other hand, DDRX has clear nucleation and growth stages [29,37].

Recent studies have provided important vision into the mechanisms of DRX in the magnesium alloys. The mechanisms of DRX are dependent on the deformation temperature and strain rate [38]. During FSP, the material flow is driven by the rotating pin, and the strain rate ( $\overset{•}{\varepsilon }$) can be derived by a torsion-typed deformation as [22]:

$$\overset{•}{\varepsilon }=\frac{R_{m}\cdot 2\pi r_{e}}{L_{e}}$$ (3)

where *R_m_* is about half of the pin rotation speed, *r_e_* and *L_e_* are the effective radius and depth of the dynamic recrystallized zone. As the NFSP and SFSP materials in the SZ undergo the plastic flow, the value of *r_e_* and *L_e_* can be determined by observing the boundary of SZ according to the Ardell *et al*. [39] analysis. In the current work, the observed zone boundary radius is 1.69 mm and 1.58 mm for the NFSP and SFSP specimens, and the value of *L_e_* is 2.34 mm (Figure 2). For the given *R_w_* of 900 rpm, can be calculated to be 26.5 s^−1^ and 24.8 s^−1^ for NFSP and SFSP. As a soften mechanism for material deformation, DRX takes place to compensate strain hardening under such high strain rate. The apparent strain softening behavior can be observed during hot deformation due to DRX [40,41]. Moreover, as the strain rate of SFSP is nearly equal to NFSP, their DRX mechanisms are mainly influenced by deformation temperature. The deformation temperatures of the two FSP procedures are quite different, and the average peak temperature is 327 °C and 215 °C, respectively (Figure 1). Sitdikov *et al.* [42] proposed that CDRX operated in the establishment of steady-state flow in the ranges of intermediate and high temperature, while DDRX played a minor role for pure magnesium. CDRX was thought to work at between 200 °C and 300 °C and high strain rates, while CDRX and DDRX may be responsible for recrystallization above 300 °C [43]. As a matter of fact, during thermo-mechanical treatments, such as ECAP, rolling, or extrusion, CDRX is, thus, considered to be the main mechanism of grain refinement [22]. The fast diffusion rate of magnesium alloys at elevated temperature of 200–400 °C also favors CDRX [44].

In our present study, for the NFSP specimen, the average peak temperature (327 °C) is in the range of high temperature, indicating both CDRX and DDRX may operate. As shown in Figure 6a,b, dislocations can be observed in the recrystallized grain interior as well as along the grain boundaries. Furthermore, the serrated grain boundary and fine subgrains can be clearly recognized. Serrated boundaries occur when the density of dislocations entering the grain boundaries exceeds their absorption capacity or when the process of lattice dislocation absorption requires an incubation time [45]. When excessive lattice dislocations pile up and generate a local stress concentration on the grain boundary, serrated boundaries will be formed. To decrease the stress concentration, the pile-up dislocations need to rearrange to form dislocation cell structures. Meanwhile, nuclei are readily formed during DRX in regions with the highest local degree of deformation, such as grain boundaries and deformation bands. However, the coarse microstructures in the BM can not provide enough such site, thus, more dislocation walls and subgrain boundaries are formed to accommodate the high strain incompatibility. Based on the measured temperature and microstructure observation, CDRX may be reasonable for the grain refinement in the NFSP specimen. On the other hand, the measured deformation temperature (215 °C) during SFSP belongs to the range of intermediate temperature (200–300 °C), and the strain rate is relatively high. TEM images showed that dislocations and subgrain boundaries are observed in the SFSP recrystallized grains. Some “necklace” structures, which generally formed in the initial stage of CDRX [43], are also visible in the TMAZ (Figure 5). Compared with SZ, TMAZ experiences lower temperature and lower deformation during FSP. Therefore, microstructure evolution in the TMAZ can be considered as the initiation of DRX. Both the theoretical prediction and microstructure observation indicate that the mechanism of DRX may be associated with CDRX for the SFSP specimen. With the same BM and the same DRX mechanism, the grain size difference between NFSP and SFSP may be attributed to the kinetics of grain growth during these two processes. According to Equation (2), due to the newly-formed grains growing at lower temperature with shorter time, finer grain size is achieved in SFSP.

## 4. Experimental Section

**Figure 10.**
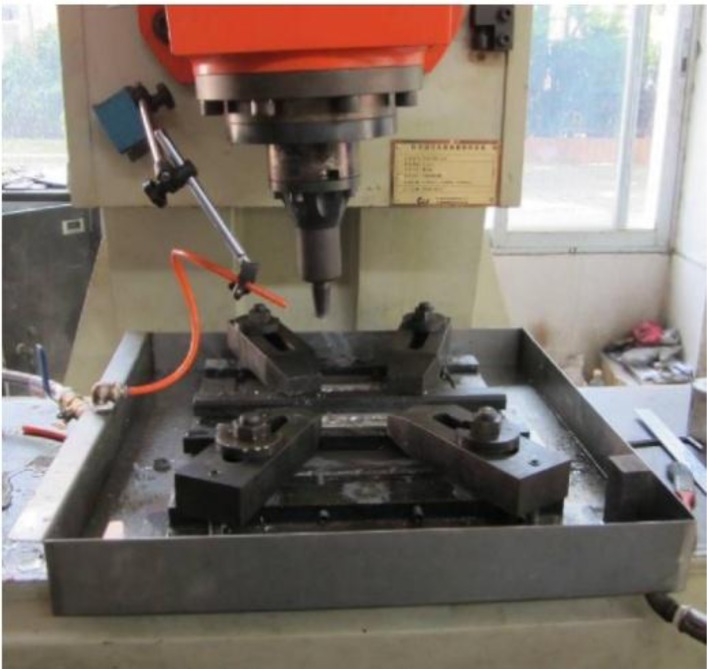
Photograph of the SFSP equipment.

The base material (BM) was hot-rolled AZ31 magnesium alloy plate with a thickness of 4 mm. The chemical compositions and mechanical properties were listed in Table 2. FSP experiments were carried out on FSW-RT31-003 welding machine (FSW Technology Co. Ltd., Beijing, China) with a 3 mm diameter, 3 mm length cone-threaded pin, and a concave shoulder 10 mm in diameter. NFSP and SFSP were performed parallel to the rolling direction of the plate with a tool rotation rate of 900 rpm and a processing speed of 60 mm/min. The FSW machine equipped with a water cooling device was used for SFSP, and the photograph of the equipment was shown in Figure 10. Before SFSP, water at room temperature was filled into the tank to submerge the plates. During SFSP, the flow rate of the water was kept constant as 29 mL/s.

**Figure 11.**
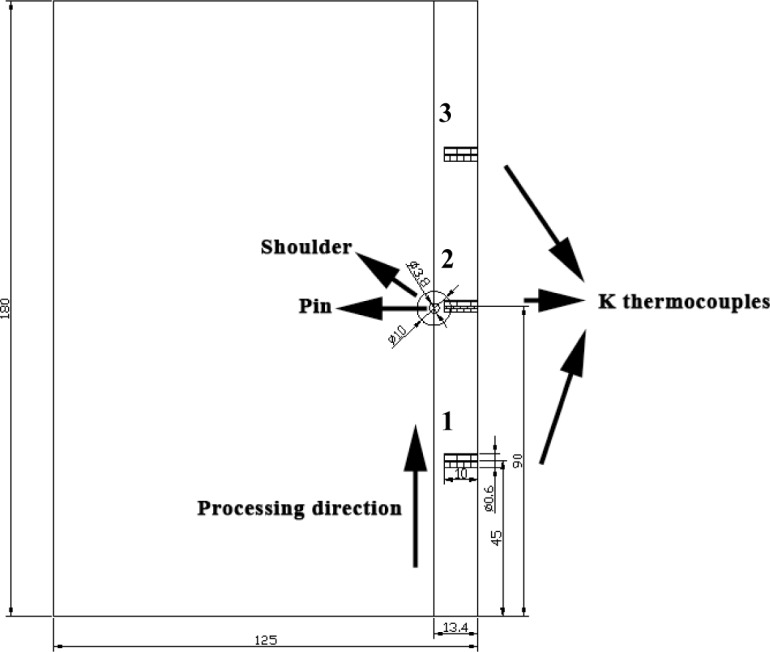
Schematic diagram of positions for measurement of temperature (Unit: mm).

Figure 11 shows the schematic diagram of positions for measurement of the temperatures. K-type thermocouples with a diameter of 0.5 mm were inserted into the middle of the plates to measure the temperatures during FSP. The measured locations were 1.5 mm distance to the edge of the pin. A Kistler CoMo injection-type device (Kistler Instrument AG, Winterthur, Switzerland) was used to continuously record the temperature history at a sampling rate of 0.25 s. The temperature measurements were repeated five times to achieve a reasonable degree of accuracy.

The specimens used for microstructure examinations were cross-sectioned perpendicular to the FSP direction. Microstructure observation and analysis were observed by LEICA optical microscopy (OM) (Leica, Wetzlar, German), Nova Nano 430 scanning electron microscopy (SEM) (FEI, Hillsboro, TX, USA), and transmission electron microscopy (TEM) (JEOL, Tokyo, Japan). The specimens for OM and SEM observation were prepared by mechanical polishing and etching using a solution of 5 g picric acid + 10 mL acetic acid + 10 mL water + 80 mL ethanol. Thin foils with a thickness of about 0.4 mm were prepared for TEM specimens. After being mechanically ground to approximately 80 μm, the foils were further ground to a thickness 25 μm by a Gatan-656 dimple grinder (Gatan Inc., Pleasanton, CA, USA). Final thinning of the foils for TEM was conducted by ion-milling technique operated at 5 kV. TEM observation was carried out on a JEM-2200FS TEM (JEOL, Tokyo, Japan) at 200 kV. Microhardness profiles were performed on a HVS-1000 digital hardness tester (Hengxinjie Technology Co., Ltd, Shenzhen, China) with a load of 500 g and a dwelling time of 20 s. The FSP tensile specimens with a gauge dimension of 3 mm × 2.5 mm × 1.5 mm were cut by electro-discharged machining parallel to the processing direction so that the gauge parts consisted of the stir zone (SZ) only, and tensile tests were performed on a SANS CMT5105 machine (SANS Co. Ltd., China, Shenzhen, China) at a strain rate of 5 × 10^−3^ s^−1^. Tensile fracture morphologies were observed by Quanta 200 SEM (FEI, Hillsboro, TX, USA).

## 5. Conclusions

In the present study, the hot-rolled AZ31 magnesium alloy is subjected to NFSP and SFSP. Thermal history of these two processes is measured, and its effect on microstructure and mechanical properties of the experimental materials is investigated. The results of the study can be summarized as follows:

(1) Due to the enhanced cooling effect of water, the peak temperature of SFSP is 100 °C lower than that of NFSP. The peak temperature drops down to ~50 °C rapidly in SFSP, indicating a higher cooling rate is achieved;
(2) SFSP is an effective method of grain refinement, and the average grain size of the NFSP and SFSP alloy is 2.9 μm and 1.3 μm, respectively. The mechanism of DRX is associated with CDRX, both for the NFSP and SFSP, and the finer grain size of SFSP is attributed to slower grain growth at lower temperature with shorter time;
(3) Mechanical properties of the SFSP AZ31 magnesium alloy are much higher that those of the NFSP alloy, especially the ductility. The SFSP AZ31 magnesium alloy shows excellent formability with an elongation of 50.5%, which is twice higher than that of the BM.

## Figures and Tables

**Table 1.** Microstructure and mechanical properties of AZ31 magnesium alloys prepared by friction stir processing (FSP).

Base material	Rotation speed, rpm	Processing speed, mm/min	Grain size, μm	Hardness, HV	Ultimate tensile strenth, MPa	Elongation, %	Reference
cast AZ31	950	30	4.2	82	206	9.1	[11]
hot-rolled AZ31	1500	60	11.4	58	223	28.4	[12]
AZ31B	180	90	2.4	85	–	–	[13]
rolled AZ31B	1500	120	3.1	–	207	44.7	[14]
AZ31B	1200	635	3–4	72	–	–	[15]
AZ31	1500	50	12.9	60	–	–	[16]
extruded	1800	160	~4.9	–	~180	~48	[17]
AZ31	800	45	2–4	69	204	19	[18]

**Table 2.** Chemical compositions (mass fraction, %) and mechanical properties of AZ31 alloy.

Chemical compositions (wt%)							Mechanical properties	
Al	Zn	Mn	Si	Cu	Fe	Mg	Tensile strength	Elongation
2.57	0.84	0.32	0.03	0.003	0.006	Bal.	~250 MPa	~25%
